# Supplementary material for: Computed Tomography Texture Analysis of Carotid Plaque as Predictor of Unfavorable Outcome after Carotid Artery Stenting: A Preliminary Study
Source: Diagnostics (Basel). 2021 Nov 27;11(12):2214. doi: 10.3390/diagnostics11122214 (PMC8699962; doi:10.3390/diagnostics11122214)
Supplement: Supplementary file 1 [file diagnostics-11-02214-s001.zip › diagnostics-1409938-supplementary.pdf]

International Journal of
Molecular Sciences

MDPI

Table S1. Complete demographics, clinical, anatomical, and procedural carotid artery stenting findings.

Variables	Overall (n=172)	Good outcome (n=155)	Unfavorable outcome (n=17)	*p*-value
Gender<br>• male<br>• females	 112/172 (65%)<br>60/172 (35%)	 100/155 (65%)<br>55/155 (35%)	 12/17 (70%)<br>5/17 (30%)	0.790
Age	77 (70-82)	77 (70-82)	79 (72-83)	0.243
Smoking history (current or former; n=90)	64/90 (71%)	56/81 (69%)	8/9 (89%)	0.438
Ipsilateral neurological ischemic event within 6 months before CAS	90/172 (52%)	83/155 (53%)	7/17 (41%)	0.444
Cardiac disease	71/172 (41%)	60/155 (38%)	11/17 (64%)	0.066
LDL (mg/dl) within 180 days before CAS	116 (97-133)	116 (96-134)	106 (105-129)	0.895
Hypertension	109/172 (63%)	101/155 (65%)	8/17 (47%)	0.185
Diabetes	53/172 (30%)	47/155 (30%)	6/17 (35%)	0.782
Arch type II-III	66/172 (38%)	63/155 (40%)	3/17 (17%)	0.071
Bovine arch	51/172 (30%)	43/155 (27%)	8/17 (47%)	0.158
Pre-dilatation	18/172 (10%)	18/155 (11%)	0/17 (0%)	0.221
Procedural time (min)	18 (15-22)	18 (16-22)	15 (15-20)	0.098
Post-procedural hypotension	11/172 (6%)	9/155 (5%)	2/17 (11%)	0.297
Post-procedural complications<br>• overall<br>• vascular access pseudoaneurysm<br>• acute stent thrombosis	6/172 (4%)<br>3/172 (2%)<br>3/172 (2%)	4/155 (2%)<br>3/155 (1%)<br>1/155 (1%)	2/17 (11%)<br>0/17 (0%)<br>2/17 (11%)	0.108<br>1.000<br>0.026

Categorical data are showed as number and percentage in parenthesis. Continuous variables are presented as median with interquartile range in parenthesis. Abbreviations: CAS, carotid artery stenting; LDL, low-density lipoprotein.

Table S2. Complete CTA features, plaque visual assessment and texture parameters.

**Variables**	**Overall (n=172)**	**Good outcome (n=155)**	**Unfavorable outcome (n=17)**	***p*-value**
Time between CTA and CAS (days)	25 (6-102)	27 (6-100)	22 (8-124)	0.961
Scanner				
• 64-rows (Toshiba)	70/172 (41%)	64/155 (41%)	6/17 (35%)	0.796
• 16-rows (Siemens)	46/172 (26%)	42/155 (27%)	4/17 (24%)	1.000
• 16-rows (Philips)	56/172 (33%)	49/155 (32%)	7/17 (41%)	0.425
Side				
• right	92/172 (53%)	81/155 (52%)	11/17 (64%)	0.443
• left	80 (47%)	74/155 (48%)	6/17 (36%)	
Visual plaque classification				
• mixed	166/172 (96%)	150/155 (97%)	16/17 (94%)	0.469
• non-calcified	6/172 (4%)	5/155 (3%)	1/17 (6%)	
Visual soft component pattern				
• homogenous	84/172 (48%)	73/155 (47%)	11/17 (64%)	0.205
• heterogenous	88/172 (52%)	82/155 (53%)	6/17 (36%)	
Napkin-ring sign	17/172 (10%)	14/155 (9%)	3/17 (17%)	0.765
Plaque ulceration	58/172 (33%)	48/155 (30%)	10/17 (58%)	0.029
Ostial plaque	91/172 (52%)	83/155 (53%)	8/17 (47%)	0.620
Angiographic stenosis (%)	68 (60-75)	69 (60-75)	64 (51-74)	0.232
Plaque length ≥15 mm	85/172 (49%)	80/155 (51%)	5/17 (29%)	0.123
Recurrent plaque	18/172 (10%)	16/155 (10%)	2/17 (11%)	0.693
Plaque volume (ml)	109 (72-160)	114 (74-161)	82 (67-156)	0.467
Plaque mean density (HU)	225 (146-353)	222 (144-349)	244 (158-389)	0.570
Plaque standard deviation density (HU)	229 (142-340)	228 (141-336)	254 (146-365)	0.713
Plaque kurtosis	5.75 (3.91-9.31)	5.84 (3.96-9.97)	5.37 (3.27-6.32)	0.048
Plaque skewness	1.63 (1.13-2.26)	1.67 (1.14-2.36)	1.53 (0.96-1.68)	0.093

Categorical data are showed as number and percentage in parenthesis. Continuous variables are presented as median with interquartile range in parenthesis. Angiographic stenosis assessment was based on North American Symptomatic Carotid Endarterectomy Trial (NASCET) criteria [28]. Abbreviations: CAS, carotid artery stenting; CTA, computed tomography angiography; HU, Hounsfield units.

Table S3. Logistic regression analysis for the relationship between clinical, anatomical and textural plaque features to predict unfavorable outcome after carotid artery stenting (n=172).

**Variables**	**Univariable analysis**			**Multivariable analysis without textural features**			**Multivariable analysis with textural features**		
	**Coefficient**	**OR (95%CI)**	***p*-value**	**Coefficient**	**OR (95%CI)**	*p*-value	Coefficient	OR (95%CI)	*p*-value
Age	0.04	1.04 (0.97-1.12)	0.188	-	-	NS	-	-	NS
Gender (male as reference)	-0.27	0.75 (0.25-2.26)	0.618	-	-	NS	-	-	NS
Cardiac disease	1.06	2.91 (1.01-8.26)	0.045	1.09	3 (1.03-8.71)	0.042	1.11	3.05 (1.02-9.09)	0.045
Diabetes	0.22	1.25 (0.43-3.58)	0.673	-	-	NS	-	-	NS
Neurological symptoms within 6 months before CAS	-0.49	0.61 (0.21-1.67)	0.336	-	-	NS	-	-	NS
Arch type II-III	-1.16	0.31 (0.08-1.13)	0.077	-	-	NS	-	-	NS
Bovine arch	0.83	2.31 (0.83-6.39)	0.105	-	-	-	-	-	NS
Plaque ulceration	1.15	3.18 (1.14-8.86)	0.026	1.19	3.28 (1.16-9.31)	0.025	1.37	3.96 (1.34-11.72)	0.012
Ostial plaque	-0.25	0.77 (0.28-2.1)	0.611	-	-	NS	-	-	NS
Recurrent plaque	0.14	1.15 (0.24-5.53)	0.853	-	-	NS	-	-	NS
Plaque length ≥15 mm	-0.94	0.39 (0.13-1.16)	0.091	-	-	NS	-	-	NS
Plaque volume (ml)	-0.00017	0.99 (0.99-1.00)	0.613	-	-	-	-	-	NS
Plaque mean density (HU)	0.00048	1.00 (0.99-1.00)	0.745	-	-	-	-	-	NS
Plaque standard deviation density (HU)	0.00064	1.00 (0.99-1.00)	0.751	-	-	-	-	-	NS

Plaque kurtosis	-0.19	0.82 (0.68-0.99)	0.043	-	-	-	-0.22	0.79 (0.65-0.97)	0.029
Plaque skewness	-0.60	0.54 (0.27-1.07)	0.079	-	-	-	-	-	NS

Abbreviations: CAS, carotid artery stenting; CI, confidence interval; HU, Hounsfield units; NS, not significant; OR, odds ratio.
